# Exploring the treatment burden of disease-modifying anti-rheumatic drug monitoring in people with rheumatoid arthritis

**DOI:** 10.1093/rap/rkad054

**Published:** 2023-06-16

**Authors:** Sarah Ryan, Laurna Bullock, Fay Manning, Carolyn A. Chew-Graham, Zoe Paskins

**Affiliations:** Haywood Academic Rheumatology Centre, Midland Partnership University NHS Foundation Trust, Haywood Hospital, Stoke on Trent, UK; School of Nursing and Midwifery, Keele University, Keele, UK; School of Medicine, Keele University, Keele, UK; School of Medicine, University of Exeter, Exeter, UK; School of Medicine, Keele University, Keele, UK; Haywood Academic Rheumatology Centre, Midland Partnership University NHS Foundation Trust, Haywood Hospital, Stoke on Trent, UK; School of Medicine, Keele University, Keele, UK

**Keywords:** RA, DMARDs, drug monitoring, treatment burden, qualitative methods

## Abstract

**Objectives:**

People with RA taking DMARDs require safety monitoring to identify potential side effects. The aim of this study was to explore the perspectives of patients and family members on DMARD monitoring and how the associated treatment burden could be minimized to optimize concordance and safety.

**Methods:**

Thirteen adults with RA on DMARDs and three family members participated in semi-structured telephone interviews between July 2021 and January 2022. Data were analysed using a framework method. Findings were discussed with a group of stakeholders to develop implications for practice.

**Results:**

Two main themes were identified: (i) making sense of drug monitoring; and (ii) work involved in drug monitoring. Participants perceived DMARDs as necessary to reduce symptoms, with drug monitoring providing an opportunity for a holistic assessment of wellbeing. Participants expressed a preference for face-to-face consultations, which allowed them to share their concerns, rather than remote, often transactional, care. The limited availability of convenient appointment times, travel requirements and parking increased the work involved for patients and family members.

**Conclusion:**

Drug monitoring was accepted as a necessity of DMARD treatment, but increased the work for people with RA related to organizing and attending appointments. The potential for treatment burden needs to be assessed proactively by clinicians when a DMARD is commenced. Where identified, strategies for minimizing the treatment burden can form part of a shared management plan, including the offer of regular contact with health professionals, with an emphasis on person-centred care.

Key messages

- Patients viewed DMARDs as a necessity to reduce symptoms and disease activity so accepted the need for drug monitoring.
- Lack of flexibility with appointments, travel requirements and parking increased the work of DMARD monitoring.
- The potential for treatment burden should be considered when DMARDs are commenced.

## Introduction

Treatment burden refers to the work that patients with a long-term condition have to perform in response to the requirements of their health-care providers and the impact that these practices have on their wellbeing [1]. The key components of treatment burden are learning about treatments and their consequences, engaging with others, concordance to treatments, and lifestyle changes and monitoring of treatment [1]. If not addressed, treatment burden has the potential for serious consequences for the patient, including poor concordance to prescribed treatments, poor clinical outcomes and ineffective use of health-care resources [2].

In RA, DMARDs minimize symptoms and slow disease progression. Despite the benefits of DMARDs, their use poses potential threats to patient safety, including hepatotoxicity, leucopenia and impaired kidney function. Consequently, patients require blood monitoring, and for some DMARDs, urinalysis, blood pressure and weight are also evaluated in accordance with national guidelines [3]. In most rheumatology services in the UK, DMARD therapy is initiated by a rheumatologist or specialist rheumatology nurse.

Although treatment burden has been identified as a concern in other long-term conditions, the potential impact on people with RA has not been investigated. We aimed to explore the perspectives of people with RA and family members on safety monitoring for DMARDs and to co-design recommendations on how any associated treatment burden could be minimized to optimize concordance with DMARDs and wellbeing.

## Methods

This study used qualitative methods, with semi-structured interviews to explore the perspectives of people with RA and their family members of experiences of DMARD drug monitoring, and with a stakeholder discussion group to co-design recommendations for practice. The reporting of this study is based on the Consolidated Criteria for Reporting Qualitative Health Research [4]. Ethical approval was granted by Surrey Research Ethics Committee REC reference 21/PR/0533. Participant consent was obtained before the interviews.

### Recruitment and sampling

People with RA who were taking a DMARD that required monitoring were recruited from two rheumatology departments and one general practice in the Midlands, UK. Potentially eligible patients were identified by clinicians from clinical databases. Purposive sampling included age, biological sex and ethnicity. Eligible patients were posted an invitation letter, expression of interest form and participant information sheet. When an expression of interest form was received by the research team, a consent form was posted or emailed, depending on participant preference, by the research team. All participants gave written consent. Family members identified during interviews with patients were invited to participate in an interview.

### Data collection

Semi-structured telephone interviews were conducted by L.B. (an experienced qualitative researcher) between July 2021 and January 2022. The interviewer was not previously known to the participants. The topic guides (see Supplementary Data S1, available at *Rheumatology Advances in Practice* online) focused on the experience of DMARD monitoring from the perspective of the person with RA and their family member and were modified iteratively as data generation and analysis occurred. The interviews were recorded digitally.

Data saturation for patient participants occurred after the 11th interview. Owing to the low number of family members recruited, data saturation was not achieved for this dataset.

### Data analysis

Data were analysed using a framework method [5]. Each interview was transcribed verbatim. Two researchers (L.B. and S.R.) were involved in the initial coding and development of an analytical framework. The framework was informed by the theory of treatment burden and by the necessity–concerns theoretical model, in which the decision to commence medication is influenced by beliefs about the necessity of treatment balanced with concerns about taking the medication [6]. All members of the research team were involved in grouping codes into themes and categories, and public contributors assisted with interpretation of the data. See Supplementary Data S2, available at *Rheumatology Advances in Practice* online, for more detail of the stages involved in data analysis.

### Patient and public involvement and engagement

A patient advisory group (PAG), consisting of four public contributors with RA on DMARDs, met three times during the study to share examples of treatment burden, to develop the topic guide and public-facing information and to facilitate interpretation of the data.

### Co-design of recommendations

A stakeholder group was convened to discuss the findings and co-design recommendations for DMARD monitoring to address the treatment burden identified. Clinical and academic representatives were invited from personal networks of the study team, while public contributors were members of the patient and public involvement and engagement group.

Stakeholders were presented with extracted quotes describing the work that people with RA and their family members undertook to engage in drug monitoring. Recommendations for practice were derived from discussions with stakeholders and the PAG.

## Results

Sixteen interviews were conducted with 13 people with RA and three family members. Twelve people with RA were recruited from two hospital rheumatology departments, and one person was recruited from a general practice. Participant characteristics are given in Table 1.

**Table 1:** Participant demographics

Characteristic	Patient participants (PwRA)				Family members (FM)
Sex	Females	10	Males		3
	Males	3			
Diagnosis	RA	13			
Age, years	40–49	3	70–79		3
	50–59	3			
	60–69	3			
	70–79	4			
Disease duration	1 month–5 years	2	Not applicable		
	6–10 years	2			
	11–15 years	3			
	16–20 years	1			
	≥21 years	5			
Occupational status	Retired	7	Retired		3
	Working	2			
	Not currently working	4			
Site of drug monitoring	Hospital site 1	10			
	Hospital site 2	2			
	General practice	1			

Hospital site 1 is North Staffordshire. Hospital site 2 is West Midlands.

The interviews lasted between 38 and 100 min (mean duration 78 min). Two main themes were identified: (i) making sense of drug monitoring; and (ii) work involved in drug monitoring. Illustrative data are provided with each data extract, labelled to indicate either a person with RA (PwRA) or a family member (FM). Ten people, from England, attended an online stakeholder meeting, including three rheumatology nurses, three clinical academics, three doctors (two rheumatologists and one general practitioner) and one person with RA.

### Making sense of DMARDs and drug monitoring

#### Understanding the need for DMARDs

For people with RA and their families, taking DMARDs was a necessity for reducing symptoms to improve function.

So I know for a fact that without medication I don’t think I could actually get out of bed. (P3, PwRA, female, 58 years old)

It can only be a couple of weeks of not having the injections and she really starts to suffer. (P14 FM, male, 74 years old)

The need for DMARD treatment outweighed concerns about side effects.

I’m happy to stay on the methotrexate despite the side effects, because it works for my rheumatoid arthritis. (P8, PwRA, female, 58 years old)

##### Recognizing the need for drug monitoring

There was recognition that drug monitoring was a requirement of taking DMARDs in order to identify any potential problems related to the medication.

You were going to have a blood test, that was kind of reassuring, even if I didn’t think there was a problem. If there was, it could be quickly identified and something done. (P12, PwRA, female, 71 years old)

It’s a bit of inconvenience isn’t it? But it’s only for my own good. It’s what has to be done, so I’ll just go along with it. (P16, PwRA, female, 65 years old)

##### Expectations of drug monitoring consultations

Many participants found remote engagement with health professionals via telephone advice lines acceptable for reporting DMARD side effects but would prefer a face-to-face consultation for discussing concerns about their condition.

When my hair was coming out, I rang the helpline and they came back to me … I know they’re always at the end of the ’phone if I’ve got any concerns. (P11, PwRA, male, 76 years old)

I think when you've got a fluctuating condition, having that security net of being able to see the monitor nurse is a good thing to have. I can’t seem to sort out problems over the ’phone. (P8, PwRA, female, 58 years old)

Some participants perceived that drug monitoring was primarily focused on having a blood test and missed the opportunity of being able to discuss their concerns.

I have the blood tests, and as long as it was OK then that’s it. Whereas before, we could see one of the nurses, and that was useful if you wanted to say, ‘I’m not good’. (P9, PwRA, female, 74 years old)

### The work involved in drug monitoring

#### Impact on the person with RA

The nature of the work involved in drug monitoring impacted on the person with RA and family members. For the person with RA, the work included making appointments, travelling to and parking at clinics, and obtaining blood results to determine whether they could continue with treatment.

It isn’t that easy to book in blood tests because they do fill up very, very quickly. (P14, FM, male, 74 years old)

At one point I was going for bloods every 2 weeks, and it is 20–30 min drive. Obviously, you’ve got the parking costs. (P6, PwRA, female, 45 years old)

Getting results from blood tests can be difficult, contacting someone in the hospital to find out if we can go ahead with the injection or not. (P10, FM, male, 72 years old)

This work was exacerbated when appointment times did not accommodate the physical challenges experienced by people with RA.

Anybody with rheumatoid arthritis does not want to have to get up 3 h before an appointment so that they can move enough to get out of the house. (P3, PwRA, female, 58 years old)

Treatment burden may be greater for people who are working and reliant on the support of their employer to be able to attend for drug-monitoring appointments.

If you were doing a job, you’re relying on that employer to be understanding. (P6, PwRA, female, 45 years old).

I had to go to the hospital, and that was a bit inconvenient because I was working. (P5, PwRA, male, 67 years).

##### Impact on family members

Work for family members centred on supporting appointment making and attendance. Although the involvement of a family member could reduce the work involved for the person with RA, it increased the burden on the family.

I always took her there anyway because it’s easier. I’d drop her off, then I’d wait outside if she was just going for a blood test. (P15, FM, male, 74 years old)

Well, my husband says he’ll take me [to appointments], but obviously, it’s difficult for him because he’s working, so it’s pressure on him. I think it’s a knock-on effect for the family. (P6, PwRA, female, 45 years old).

### Stakeholder discussions

The findings from the interviews and the PAG meetings were shared with the stakeholder group. This provided the opportunity for clinician and patient representatives to place the study findings into their own context and develop recommendations for how the treatment burden could be identified and addressed within current consultations. Participating rheumatologists described uncertainty regarding whether treatment burden was discussed and, if so, by which health professionals. Clinicians perceived that identifying the potential for treatment burden often occurred reactively, when patients failed to attend for drug monitoring or developed additional health conditions, rather than proactively at the commencement of DMARD treatment. It was suggested that identifying the treatment burden during telephone consultations might be more challenging than in a face-to-face situation. Core recommendations for addressing the treatment burden in practice, co-designed by the stakeholders, PAG and the research team included: (i) conceptualizing monitoring as person centred; (ii) proactively identifying the treatment burden; and (iii) minimizing the treatment burden in partnership with the person with RA.

## Discussion

This qualitative study explored the treatment burden associated with DMARD monitoring in people with RA. Key findings were that taking DMARDs and subsequent engagement in drug monitoring was a necessity to reduce disease activity. Concerns about potential side effects were accepted in return for control of symptoms. Conceptualizing monitoring as drug monitoring was a misnomer because the process was perceived to involve not only safety surveillance, but a more holistic assessment of the patient’s physical and emotional wellbeing. There was a preference for face-to-face drug-monitoring consultations with health professionals rather than remote contact; this facilitated an opportunity for patients to share treatment-related concerns and was perceived as valuable, despite the work involved in attending for appointments. The work for a person with RA involved managing inconvenient appointment times along with the time and cost required to travel and park at drug-monitoring locations. This work impacted on family members, who often assisted with travel arrangements. Treatment burden presented in different contexts, and unrecognized burden might be a particular concern for people who work.

The frequency of attending for monitoring varied from 2 weekly to 3 monthly depending on the DMARD prescribed and whether the patient was on stable treatment. Our findings resonate with previous literature demonstrating that people with RA were willing to accept the monitoring requirements and risk of side effects from DMARDs to remain on treatment and have their symptoms controlled [7, 8]. The necessity of DMARD treatment overrode the work involved with monitoring, in accordance with the ethos of the necessity–concerns framework [6], whereby high necessity beliefs (how necessary the person with RA believes DMARD therapy is for their condition) outweighed concerns about taking the medication or the work associated with drug monitoring.

In a qualitative synthesis, the telephone support provided by rheumatology health professionals was identified as being an important component of assisting patients in taking DMARDs [8]. Our findings identified that remote support was acceptable for accessing advice on side effects, but many patients and family members reported missing the personal nature of a face-to-face appointment, where they felt it was easier to share their problems, obtain information on their symptoms and access ongoing support.

Health-care systems create additional work for patients with long-term conditions [1]. Our findings identified that appointment times did not take into consideration the physical symptoms of RA, including the presence of early morning stiffness. Rigid health-care systems, including inflexible appointment times, have been shown to increase the work involved for people with chronic conditions [9–12]. Previous studies involving people with long-term health needs have identified travel time, arranging appointments and transportation as adding to treatment burden [13]. Many participants had co-morbidities, primarily cardiovascular and respiratory, which added to the burden of attending for additional medical appointments. No data were collected on how far participants travelled or the financial costs involved. Some participants chose to travel further to a hospital clinic to access specialist support, whereas others preferred to be monitored closer to home.

Treatment burden can go unrecognized by clinicians unless the impact of drug monitoring on the personal, work and social context of peoples’ lives is assessed [14]. Treatment burden can range from not knowing who to contact if problems with monitoring arise to being reliant on the understanding of employers to grant time off to attend appointments.

The involvement of public contributors in all stages of the study and the stakeholder group to develop recommendations for practice enhances the credibility of the findings. Although the participants in this study had wide ranges of age, disease duration and both sexes, the sample was primarily of white British, older individuals, the majority of whom were currently not working or were retired. It would be helpful to conduct further research on a more diverse sample of participants, including patients who are working, a wider representation of ethnicity and family members. Other methodological considerations include the impact of coronavirus disease, which precluded family members from attending the monitoring clinics. This altered our recruitment strategy, and we were reliant on patients to identify family members to be interviewed. A more direct approach might have enhanced family member recruitment.

To address the potential for treatment burden, health-care professionals need to understand which health-care tasks might contribute to the burden and how [15]. Core recommendations for addressing treatment burden associated with DMARD monitoring are included in Table 2, along with implementation suggestions. Our findings demonstrate that having access to flexible appointment times and monitoring locations could reduce the amount of work that patients and family members are required to do. Consequently, when commencing DMARDs, health professionals need to address the potential for treatment burden [13, 16]. Findings from the stakeholder discussion and PAG advocated focusing on person monitoring rather than drug monitoring, favouring a holistic assessment of needs, including the potential for treatment burden. Through shared decision-making, solutions to minimize treatment burden that are responsive and flexible to the needs of the person and their family members can be identified and implemented. For patients in whom the treatment burden is a major concern, periodic face-to-face consultations might be beneficial to assess whether the work required from the patient and or family member remains manageable.

**Table 2:** Core recommendations for addressing treatment burden associated with DMARD monitoring

Core recommendation	Action	Implementation suggestions
Conceptualize monitoring as person centred	Change the emphasis of care from drug monitoring to person monitoring to facilitate a holistic assessment of the person and their condition	Discuss with the person what drug monitoring is and what it involves Describe drug monitoring in patient-facing materials as an integrated component of person-centred ongoing care
Proactively identify the treatment burden	Identify which person within the health-care team is responsible for identifying the treatment burden and when. Ideally, potential for treatment burden needs to be evaluated proactively when DMARDs are commenced and reassessed over time as a person’s life circumstances might change	Discuss with the person how they feel about attending for drug monitoring and whether they foresee any difficulties in managing the requirements Ask the person to explain where and when they will have their monitoring to identify any potential problems Revisit the potential for treatment burden if the person’s context alters (e.g. new co-morbidity or change in social circumstances)
Minimize treatment burden	Negotiate with the person how treatment burden can be managed when it is identified	Offer a range of monitoring options responsive to the person’s identified needs Where treatment burden is evident, offer additional support (e.g. monthly telephone review) to evaluate how the burden is being managed Consider the use of peer patient support, meaning that people can learn from others how challenges with drug monitoring might be overcome

## Conclusions

Drug-monitoring services should be focused around the person, with the potential for treatment burden being proactively assessed and managed, working in partnership with people with RA and their family members. Offering flexible options, including a range of appointment times and drug-monitoring locations, could reduce the work involved for patients and their families. Further research is required to explore how best to identify and minimize the treatment burden for people with RA.

## Supplementary Material

rkad054_Supplementary_Data

## Data Availability

The data underlying this article are available in the article and in its own online supplementary material.
