# Supplementary material for: Exploring the treatment burden of disease-modifying anti-rheumatic drug monitoring in people with rheumatoid arthritis
Source: Rheumatol Adv Pract. 2023 Jun 16;7(2):rkad054. doi: 10.1093/rap/rkad054 (PMC10307943; doi:10.1093/rap/rkad054)
Supplement: rkad054_Supplementary_Data [file rkad054_supplementary_data.docx]

**SUPPLEMENTARY DATA S1: Topic Guides.**

**Topic Guide for the Person with RA.**

**Introduction to the Study.**

**Demographic Questions:**

- Age
- Gender
- DMARD use
- Whether they usually attend accompanied or unaccompanied

1. Can you tell me how long you have had RA?
2. What medicines do you take for your RA?
3. Can you tell me why you agreed to participate in this study?
4. How important is it to you that you take your medicines for your RA?
5. How important are the thoughts of your family and friends in taking your medicines?
6. Can you describe any concerns that you have about taking your medicines?
7. Can you describe what happens when you attend for drug monitoring?

- Where do you go?
- Who do you see (phlebotomist or nurse)?
- Do you have any additional tests other than your blood being taken e.g. blood pressure, urinalysis?
- How often do you attend?
- Do you go with anyone else such as a family member or friend? If so what is the benefit of this?
- How long does it take?

1. Why do you attend for monitoring?

- What motivates you?
- Do you ever not attend, why?

1. Are there any aspects of attending for drug monitoring that work well for you?
2. Can you describe any times when attending for drug monitor has seemed difficult?

- If so, has this ever affected your willingness to change your medicines, or to start a new medicine?
- Does having RA (with the potential for pain, fatigue and reduced function) ever affect where you to attend for monitoring?
- How do you balance the demands of attending for monitoring with other demands on your time?

1. Do you think the demands on you in coming for drug monitoring have changed over time?

- More or less difficult and why?

1. Have you ever not attended for drug monitoring? If not, why, and what were the consequences?
2. How could your experience of drug monitoring be improved?
3. Is there anything else you would like to add?

**Topic Guide for Family Member.**

**Introduction to the Study.**

**Demographic Questions:**

- Age
- Gender
- Whether they usually accompany x to drug monitoring

1. In what capacity do you know x?
2. Can you tell me why you agreed to participate in this study?
3. How important do you think it is for x to take tablets to help control the RA?
4. Do you have any concerns about the tablets x takes?
5. Have you ever accompanied x to a drug monitor appointment-what was that experience like? How does this impact on you?
6. Do you think it is hard work for x to attend for drug monitoring, if so why?
7. What are the challenges for you or other family members with x attending for drug monitoring?
8. Can you think of any ways that drug monitoring could be made easier/improved?
9. Is there anything else you would like to add?

**SUPPLEMENTARY DATA S2: The Stages Involved in Data Analysis (Gale et al 2013).**

**Stage 1:**

Transcription: Each interview was transcribed verbatim which enabled the interviewer to become immersed in the data.

**Stage 2:**

Familiarisation with the interviews: The interviewer listened to the digital recordings, making analytical notes and documenting thoughts or impressions related to treatment burden.

**Stage 3:**

Coding: Two members of the research team were involved in coding enabling all data to be compared systematically.

**Stage 4:**

A working analytical framework was developed which involved several meetings with the research team to agree on a set of codes to be applied to subsequent transcripts. Codes were then grouped into themes and categories to form a working framework.

**Stage 5:**

Charting data into the framework matrix: Charting involves summarising the data by category from each transcript. The working analytical framework was then applied by indexing subsequent transcripts using the existing categories and codes.

**Stage 6:**

Interpreting the data: Characteristics of and differences between the data were identified, interrogating the theoretical concepts of treatment burden as well as mapping connections between categories to explore relationships with the data.

#### Stage 7:

#### Interpreting the data: This involved all members of the research team mapping connections between categories to explore relationships and/or causality to understand the individual, family and health system associations with treatment burden.
